# CoCrMo alloy *vs*. UHMWPE Particulate Implant Debris Induces Sex Dependent Aseptic Osteolysis Responses *In Vivo* using a Murine Model

**DOI:** 10.2174/1874325001812010115

**Published:** 2018-03-30

**Authors:** Stefan Landgraeber, Lauryn Samelko, Kyron McAllister, Sebastian Putz, Joshua.J. Jacobs, Nadim James Hallab

**Affiliations:** 1 Department of Orthopaedics, University Hospital Essen, University of Duisburg-Essen, Hufelandstrabe 55, 45122 Essen, Germany; 2 Department of Orthopedic Surgery, Rush University Medical Center, Chicago, IL, U.S.A

**Keywords:** Osteolysis, Aseptic loosening, Innate immune reactivity, Implant material, Bioreactivity, Inflammatory osteolysis, Wear debris

## Abstract

**Background:**

The rate of revision for some designs of total hip replacements due to idiopathic aseptic loosening has been reported as higher for women. However, whether this is environmental or inherently sex-related is not clear.

**Objective:**

Can particle induced osteolysis be sex dependent? And if so, is this dependent on the type of implant debris (*e.g*. metal *vs* polymer)? The objective of this study was to test for material dependent inflammatory osteolysis that may be linked to sex using CoCrMo and implant grade conventional polyethylene (UHMWPE), using an *in vivo* murine calvaria model.

**Methods:**

Healthy 12 week old female and male C57BL/6J mice were treated with UHMWPE (1.0um ECD) or CoCrMo particles (0.9um ECD) or received sham surgery. Bone resorption was assessed by micro-computed tomography, histology and histomorphometry on day 12 post challenge.

**Results:**

Female mice that received CoCrMo particles showed significantly more inflammatory osteolysis and bone destruction compared to the females who received UHMWPE implant debris. Moreover, females challenged with CoCrMo particles exhibited 120% more inflammatory bone loss compared to males (p<0.01) challenged with CoCrMo implant debris (but this was not the case for UHMWPE particles).

**Conclusion:**

We demonstrated sex-specific differences in the amount of osteolysis resulting from CoCrMo particle challenge. This suggests osteo-immune responses to metal debris are preferentially higher in female compared to male mice, and supports the contention that there may be inherent sex related susceptibility to some types of implant debris.

## 1 INTRODUCTION

It is well established that long term implant loosening due to inflammatory bone loss (aseptic inflammatory osteolysis) is generally mediated by a subtle innate immune response to accumulating implant debris For some types of total hip replacement designs (*e.g*. metal on metal articulating total hip arthroplasty), there has been a reported difference in the prevalence of aseptic biologic reactivity related implant failure beteween men and women, which ranges from 30% to 100% higher in women than in men [1-7] at early time points (3-10 years). However, the reason for this is unknown and unsupported in basic science and animal models. It has been put forward that anatomical differences may be responsible for the higher failure rates in women, who general have smaller diameters of the acetabulum and the use of correspondingly smaller implants [5]. However, reports from national registry data demonstrate that sex and small diameter implants are both independent predictors for implant survival but does not account for obfuscating environmental factors, such as cosmetics exposure, diet, activity level *etc* [2]. Thus, it remains unclear if inherent sex based biologic factors affect implant performance, particularly when it comes to sex dependent effects of specific types of implant debris, such as CoCrMo implant debris from some designs of metal-on-metal (MoM) hip arthroplasty [2-4]. We hypothesized that female immune reactivity to metallic and polymeric implant debris leading to aseptic osteolysis *in vivo* (independent of environmental conditions) will be significantly higher than compared to males.

Testing our hypothesis in human cohorts is problematic since particle induced osteolysis cannot be induced and studied, and retrospecitve data is complicated by myriad environmental differences between male and female orthopaedic cohorts, such as lifestyle, body mass, implant design, surgeon-dependence, malpositioning *etc*. that may all vary based on sex. Thus, our purpose was to test this hypothesis reproducibly in a murine model of inflammatory osteolysis to examine if implant debris-induced osteolysis responses can be sex-dependent and if so, to determine the extent of bone loss induced by different types of implant debris materials (*i.e.* ultra-high molecular weight polyethylene, UHMWPE *vs*. CoCrMo particulate implant debris).

## 2 METHODS

### 2.1 Animal Model of Particle-Induced Osteolysis

We used a well-established murine calvarial model of implant debris-induced osteolysis [8, 9]. The experiments were performed on 36 (18 male / 18 female) specific-pathogen-free 12-week-old C57BL/6 wild type mice (The Jackson Laboratory, Bar Harbor, Maine, USA) in accordance with the official guidelines and were approved by the university and the local government (Rush University IACUC approval: 13-065). A 1.0 x 1.0 cm area of periosteum was exposed by making a 10 mm midline sagittal incision over the calvaria anterior to the line connecting both external ears. In the sham controls (Sham) the incision was closed without any further intervention. In the other animals the exposed periosteum was covered uniformly with 30 µl of dried pure UHMWPE polyethylene particles (Ceridust VP 3,610, Clariant, Gersthofen, Germany) or CoCrMo particles (BioEngineering Solutions Inc., Oak Park, IL). As has been reported in earlier studies, the periosteum was left intact to eliminate/minimize trauma-induced inflammation, osteolysis and osteogenesis in response to bone injury [8, 10, 11].

The particle sizes of CoCrMo alloy particles (CoCrMo, approx 60%Co, 28%Cr, <6%Molybdenum, <1%Nickel, ASTM F75) were produced from a commercially available total hip arthoplasty head component (ZimaloyTM), using proprietary cryomilling (Bioengineering Solutions Inc, Oak Park IL). Particulate size was characterized by using low angle laser light scattering (LALLS) and Scanning Electron Microscopy (SEM), Fig. (**1**). CoCrMo particles had a median diameter of 0.88 µm diameter ECD number-based (>95% less than 2µm, >80% less than 1 µm, range 0.2-11µm diameter ECD, non-Gaussian size distribution, Aspect Ratio 1.4, Granular in shape). UHMWPE particles had a median diameter of 0.95 µm diameter ECD number-based (>95% less than 3.2µm, >80% less than 1.5 µm, range 0.6-13µm diameter ECD, non-Gausian size distribution, Aspect ratio 1.2, Granular in shape). These CoCrMo alloy an UHMWPE particulate sizes and shapes of particulate debris produced from comercially available implants have been shown to be clinically relevant and able to induce inflammatory responses in innate immune cells [6, 12-14]. Subsequent to characterization particles were endotoxin cleaned using a 3 step process: 1) metal-safe detergent (Alconox), 2) sonication in 70% ethanol for 1 hr followed by a 24 hour 70% ethanol soak, and 3) a 24 hour soak in PyrocleanTM (a detergent for endotoxin removal) [15, 16]. Each step was followed by triple washing with deionized water (diH2O). Vacuum dried particles were sterilized by Ethylene Oxide or autoclaved and tested for endotoxin using a quantitative limulus assay analysis, Kinetic QCL: <0.01eu (Pyrogent 5000, Lonza). The 18 female mice were randomized equally into three groups, consisting of six mice each, receiving sham surgery (Female-Sham) or implantation of polyethylene (Female+PE) respectively CoCrMo particles (Female+CoCrMo). The 18 male mice were randomized similarly into a sham group (Male-Sham), a polyethylene group (Male+PE) or a cobalt-chromium group (Male+CoCrMo). Twelve days postoperatively, the animals were sacrificed and an elliptical plate of the calvarial caps was removed from the region between the foramen magnum, auditory canals, and orbits.

### 2.2 Micro-Computed Tomography (Micro CT)

Micro-computed tomography was used to measure the degree of osteolysis in mouse skulls. After dissection, isolated calvaria were scanned axially at 55kVp, intensity 145µA, 300-ms integration time with 30-µm isotropic voxels (Scano µCT 40, Wayne, PA, USA). To keep the samples in position during scanning, the skulls were placed in a tightly fitting rigid plastic tube filled with 10% neutral buffered formalin. To determine accurately the region of interest (ROI), a three-dimensional rendering of the whole sample was performed using the manufacturer´s software. If osteolysis or particles were visible, the ROI was defined as a cylinder with a diameter of 3.84mm and a thickness of 1.47mm beginning from the first slide that captured the bone surface in the ROI. The particle artefact was not observed and did not affect bone fraction calculations. The specific morphometric parameters bone volume (BV) and tissue volume (TV) were measured with a threshold of 350 HU (Hounsfield unit) and the BV/TV ratio was calculated.

### 2.3 Histomorphometric Analysis

Four-micrometer thick sections of calvaria were collected at the depth at which the presence of particles was detected within the calvarial tissue. The sections were mounted on glass slides and subsequently HE staining was performed. The HE-stained specimens were photographed digitally using a standard high-quality light microscope (Leica™, Wetzlar, Germany). The image was oriented with the midline suture in the middle of the field. The sections were coded and blinded prior to analysis. Histomorphometric measurements were performed with the image analysis software Aperio^®^ (Leica™, Wetzlar, Germany).

The program was calibrated using a calibration micrometer. The region of interest to be measured was defined as the area within a distance of 2 mm around midline suture. In this area the volume of the bone stock and potential osteolysis were determined and the relative ratio of osteolysis to the bone stock volume (Osteol./BV) calculated. The mean values per animal of each available section (min. 2 to max. 4 sections per animal) were calculated.

### 2.4 Statistics

Data is graphically shown as the mean with standard deviation. The Kolmogorov-Smirnov-Test was used to establish normality (p≤ 0.05) for n=18 male and n=18 female mice. ANOVA was used to establish significant differences for multigroup comparison (p<0.05). Subsequent statistical comparison was conducted with Student`s t-test where p<0.05 was used to establish significance. The software SPSS 22 (IBM, Ehningen, Germany) was used to carry out the statistical computations. Summary statistics of data are expressed as means and standard deviations.

## 3 RESULTS

**Fig. (2):**
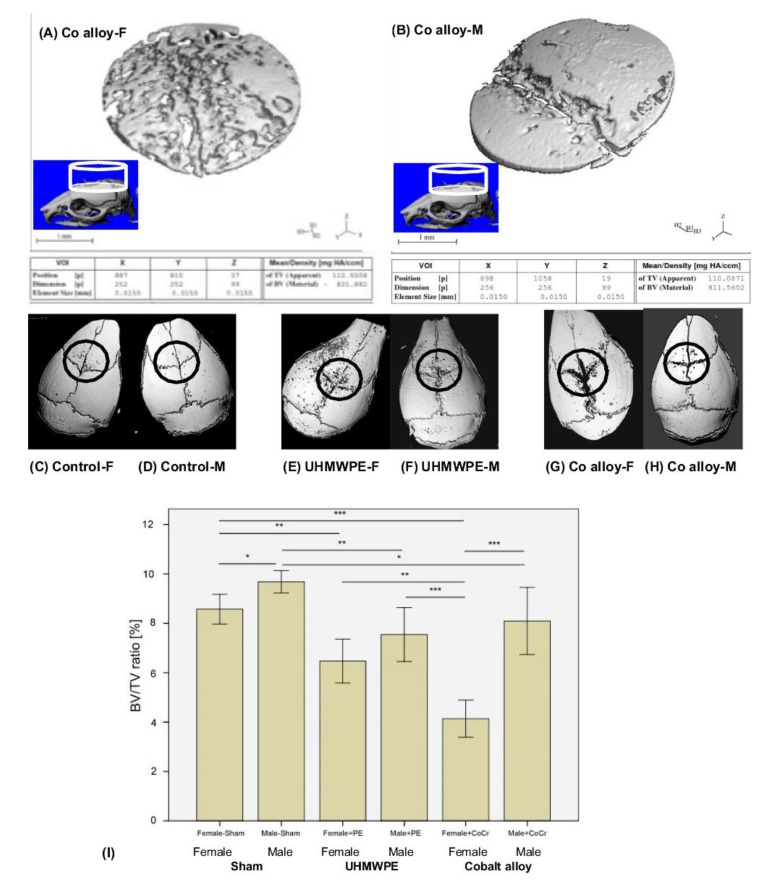
**Murine Model of Induced Osteolysis.** Examples of 3D reconstructions of bone within a standardized cylindrical volume (total volume) from uCT analysis of mouse calvaria challenged with particles of (A) CoCrMo and (B) UHMWPE showing inflammatory bone loss. (C-H) The examples of the 3D top views of mouse calvaria show the skull surface of female mice and male mice that received CoCrMo (+Co alloy) or plastic (+UHMWPE) particles or underwent sham surgery (Control). (I) Micro-CT showed pronounced bone resorption in the CoCrMo and UHMWPE particle-treated groups compared to the respective sham control group. Comparison of the particle groups with each other revealed a significantly smaller BV/TV ratio in the female mice that received CoCrMo particles than in the female mice with PE particles or particle-treated male mice (Note: Error Bars indicate standard deviations, and p values of Students T test comparison are as follows: *=p<0.05, **=<0.01, ***=<0.001).

**Fig. (3):**
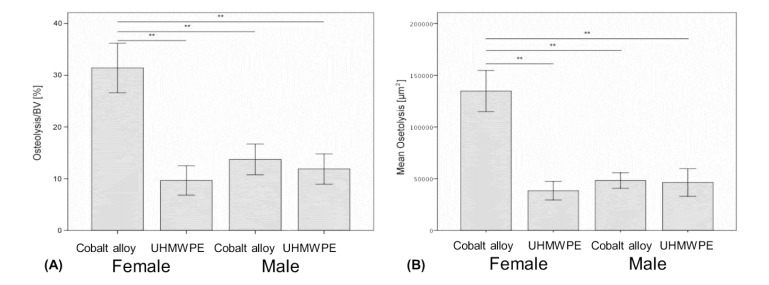
**Sex Dependent-Osteolysis to CoCr and UHMWPE.** A) Normalized to the average bone volume (BV) of sham controls, a comparison of the particle challenged groups with each other revealed a significantly larger amount of osteolysis and osteolysis/bone-volume ratio in the female mice that received CoCrMo particles than in the female mice with UHMWPE particles or particle-treated male mice. B) Normalized to the areal bone (BA) of sham controls, areal measure of osteolysis calculated by histomorphometry of the Hematoxylin Eosin (HE) stained coronal sections of calvaria at the sagittal suture showed that addition of CoCrMo induced >100% more osteolysis in female mice than male mice challenged with CoCrMo or UHMWPE particles (Note: Error Bars indicate standard deviations, and p values of Students T test comparison are as follows: *=p<0.05, **=<0.01, ***=<0.001).

We observed significant differences for all histomorphometric (osteolysis, osteol./BV) and micro-CT parameters (BV/TV) in all animals that received UHMWPE or CoCr particles on the calvaria in comparison to the sham control groups (ANOVA p<0.05, Figs. **2** and **3**).

Selected cylindrical control volumes of mouse calvaria encompassing the placement of particle challenge upon the exposed calvaria were analyzed for total bone volume and compared to sex matched controls for volume of bone loss. 3D reconstructions of bone volume (BV) within these standardized cylindrical regions of total volume (TV) were used for quantitative analysis of the murine calvaria bone loss (Fig. **2**). Example 3D reconstruction of bone within standardized regions clearly illustrates the increase in osteolysis associated with Female *vs*. Male mice challenged with CoCrMo particles (Fig. **2**). Using histomorphic measures of bone loss of bone volume (BV) to total standardized cylindrical volume (TV) of (osteolysis, osteol./BV) and micro-CT parameters (BV/TV) differences within groups were compared (Fig. **2**). Males demonstrated greater bone volume compared to females with sham surgery only, indicating slight but significant sex differences in calvaria bone volume for age matched controls. In comparison to these baseline sham controls, both metal (CoCrMo) and plastic (UHMWPE) particles induced inflammatory bone loss (p<0.05), where the greatest amount of bone loss was observed in female mice challenged with CoCrMo particles.

On a normalized basis to sham sex matched controls (osteolysis, osteol./BV), the pattern of bone loss was similar to non normalized data (Fig. **3a**). This analysis of the data revealed that females challenged with CoCrMo particles had significantly more osteolysis than did any other group with greater than 100% more bone loss than males treated with the same amount of CoCrMo particles demonstrating the severe sex effect of CoCrMo particles when compared to plastic UHMWPE particles of similar size and dose. All other challenged groups normalized to sham surgery controls (*i.e.* Females+UHMWPE, Males+UHMWPE, Males+CoCrMo) did not significantly differ. Similar results were obtained for histomorphometric (osteolysis, osteol./BV) analysis of H&E stained sections (Fig. **3b**), where there was over 120% increase (p<0.01) in the amount of areal calculated bone loss for female CoCrMo challenged calvaria compared to males with CoCrMo. Histomorphometric (osteolysis, osteol./BV) analysis also demonstrated a similar lack of sex difference between UHMWPE treated groups, supporting a metal/sex specific immunogenic mechanism of inflammatory bone loss.

**Fig. (4):**
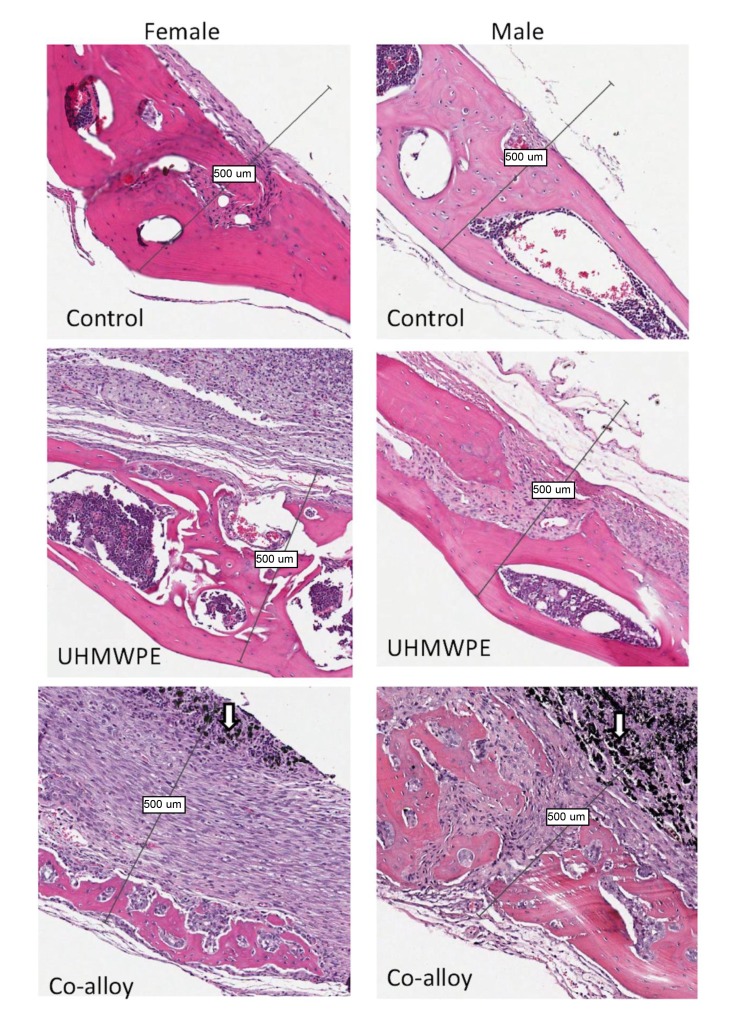
**Histological Analysis of Calvaria Tissue.** Examples of histological sections used for histomorphological calculations of bone/osteolysis area of sham, Cobalt-alloy and UHMWPE particle treated calvaria of female and male mice (arrows indicate metal particles) shows the calvaria bone destruction by invasive inflammatory tissue induced by both UHMWPE and CoCrMo particles. Representative images show the relatively larger inflammatory tissue pannus and increased bone resorption associated with CoCrMo when compared to UHMWPE particles (Note: Bars indicate approximately 0.5mm).

Over 15 histologic sections per treatment group were analyzed at the midline suture of the calvarial bone plates proximal to particle challenge used for histomorphometric analysis. Qualitative analysis revealed a relatively increased inflammatory pannus associated with CoCrMo *vs* UHMWPE in male and female mice (Fig. **4**). An inflammatory pannus with the capability to actively resorb bone is clearly illustrated where the thickness of the inflammatory tissue was approximately 2x as thick in CoCrMo challenged calvaria when compared to the pannus induced by UHMWPE for both males and females. The particle challenge is shown isolated within the inflammatory pannus and excluded from the bone interface, yet a high degree of cell infiltrates can be observed associated with pits of active bone resorption. The composition of the cell/tissue infiltrates (pannus) co-localized with pits of bone loss generally showed vascularized soft tissue fibroblasts with an abundance of macrophage or macropahge-like cells migrating into boney areas. There was no evidence of extensive lymphocyte infiltrates or aseptic lymphocyte vasculitis associated lesions (ALVAL)histology that would be associated with an adaptive immune responses. Additionally, there was no evidence of active multinuclear osteoclasts inside the calvaria responding to inflammatory stimulus and resorbing bone from the inside out (Fig. **4**). Qualitatively there was a 2x greater foreign body response associated with female murine calvaria compared to males when challenged with either UHMWPE or CoCrMo particles (Fig. **4**).

**Fig. (5):**
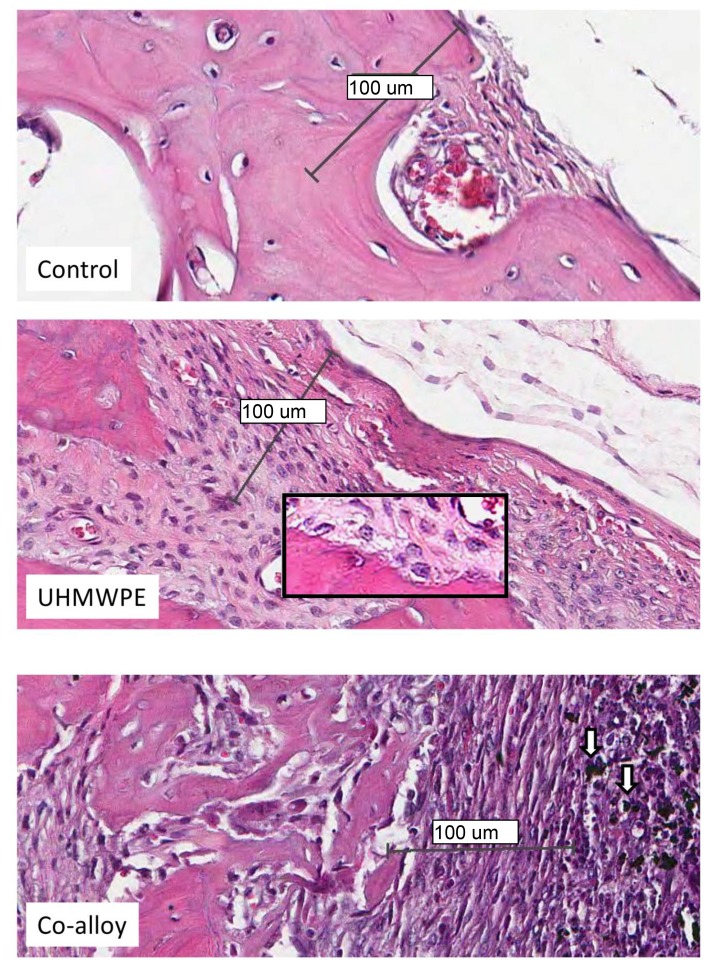
**Histological Analysis of Calvaria Tissue.** Higher magnification of histologic sections of UHMWPE and Cobalt-alloy treated female calvaria (arrows indicate metal particles) show the lack of multinucleated osteoclasts at the bone pannus interface and instead show macrophage, fibroblast and osteoclast like cells associated with bone resorbtion eminating from the inflammatory pannus. There was a notable lack of lymphocyte infiltrates associated with both CoCrMo and UHMWPE particle inflamatory tissue. Neogenic woven bone was apparent in histological sections of all particle treated calvaria (Note:Bars indicate 0.1mm).

At high magnification there was no evidence of multinucleated osteoclasts at the bone pannus interface of particle treated calvaria (Fig. **5**). Instead macrophages, fibroblasts and osteoclast-like cells were observed at the sites of bone resorption and eminating from the inflammatory pannus (Fig. **5**) (Note:Bars indicate 0.1mm). Neogenic woven bone can be seen in both UHMWPE and CoCrMo challenged calvaria (Figs. **4** and **5**) indicative of the compromise time period between maximal inflammatory osteolysis and maximal neogenic bone, used in this investigation.

## 4 DISCUSSION

Our results indicate that CoCrMo implant debris induces significantly increased osteolysis in females over that of males using a murine model of particle induced osteolysis. However, not all implant debris types induced sex based differences in osteolysis using this model. Our results indicate that UHMWPE particles do not induce significantly sex-based differences in either soft-tissue inflammation or resulting osteolysis. This is consistent with clinical observations that have reported women can suffer higher failure rates of some metal-on-metal THA designs under conditions of elevated metal release [4]. However, it still remains unclear why this occurs and there are many possible mechanisms that account for this phenomena. Previous investigations have shown that CoCrMo and polyethylene particles elicit different types of immune responses *in vitro* [17-19], which may be due to the pleotropic nature of Cobalt-alloy implant debris in general. CoCrMo implant debris can induce toxicity responses such as hypoxia [20], as well as activate innate [14, 21, 22] and adaptive immune reactivity [23-25], both *in vitro* and *in vivo*. However, UHMWPE implant particles have not been identified as inducing similar array of toxicity and immune responses, yet they do elicit innate immune reactivity inflammatory responses through inflammasome danger signaling [8, 17, 18, 26, 27]. Taken together with the current investigation, these previous reports do not support sex based differences in innate immune reactivity alone (such as NLRP3 inflammasome, TLR signaling, co-stimulatory molecule expression, cytokine expression *etc*). Instead this evidence supports a more complex picture where toxicity (**e.g*.* hypoxia, pyroptosis, necrosis, DNA damage etc) and/or adaptive immune reactivity (such as lymphocyte mediated metal hypersensitivity) [20, 23-25] may alone or in concert, produce greater downstream particle induced osteolysis responses in women. To more clearly delineate the dominant mechanisms associated with this observed sex dependence will require further investigations.

There were important limitations of using an *in vivo* murine model to approximate human particle induced osteolysis affected by sex and different implant materials. The compromise time point between maximal osteolysis and maximal bone-neogenesis, was practically limited to a single point at 12 days post-op. While this time point represents a combination of osteolysis and osteogenesis in the murine model, it precludes determination of whether the observed differences were due to sex based inflammatory osteolysis or osteogenic differences or a combination of both. Additionally, a cross sectional time of 12 days may have acted to obfuscate osteolysis differences for UHMWPE that may be significantly apparent at earlier time points, *i.e*. 5-7 days post operatively (Fig. **3**). Thus, further investigation with earlier time points and greater number of subjects may help reveal more subtle sex differences in UHMWPE. However, the results of this study support our hypothesis to some degree, indicating that sex-based differences could be detected in response to CoCrMo particulate metal debris. This is the first report demonstrating the ability of metal CoCrMo-alloy implant debris particles to induce sex dependent osteolysis differences at time points where UHMWPE did not. Because evidence for the clinical analogue of this sex-dependence has been reported for patients with metal debris in metal-on-metal hip arthroplasty revisions [28, 29], the extension of these finding to orthopedic patients is, in part, supported.

## CONCLUSION

We found sex-based influence on inflammatory osteolysis/osteogenesis reactions to CoCrMo particles (but not UHMWPE), implying increased female osteo-immuno reactivity to metallic (CoCrMo) but not polymeric (UHMWPE) implant debris. This may explain one aspect of population dependent implant performance in the outcome of orthopedic implants that preferentially release Cobalt-alloy metal debris. Further research targeting specific mechanisms of toxicity and immune responses (innate vs adaptive) that may mediate sex dependent outcomes in joint replacement surgery is needed to support these findings.

## Figures and Tables

**Fig. (1):**
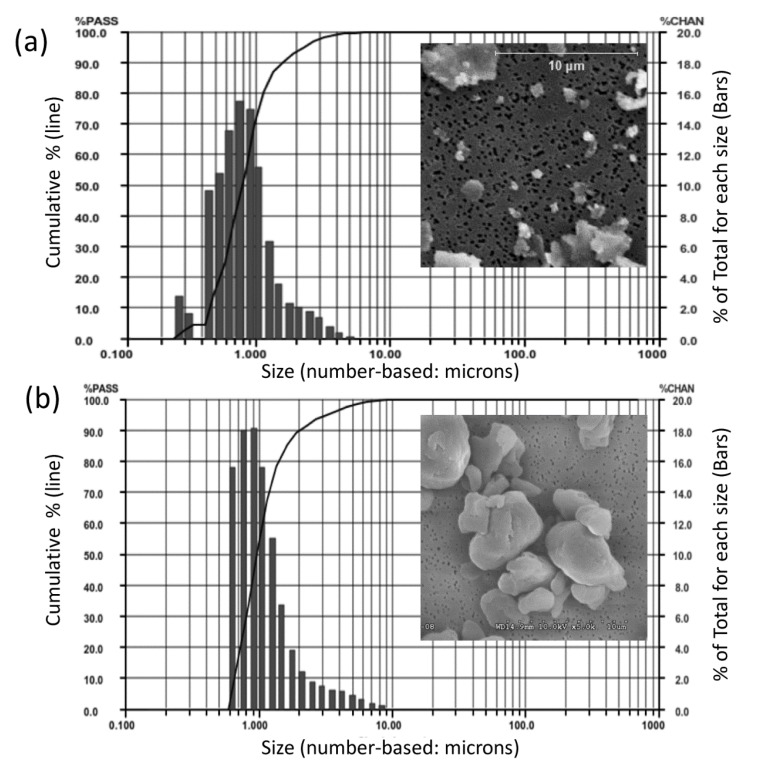
Particles were characterized by using low angle laser light scattering (LALLS) where millions of particles dispersed in liquid (double distilled water or ethanol) are counted to determine the size of (a) CoCrMo and (b) UHMWPE particles. Scanning Electron Microscopy (SEM) (insets) image analysis was used to determine size and shape (aspect ratio). CoCrMo and UHMWPE particles had median diameters of 0.88 µm and 0.95 µm ECD number-based, respectively (non Gaussian distributions). Note: Axis for % of particles in each size (bars) is on the right hand side.

## LIST OF ABBREVIATIONS

µCT: = Micro-computed Tomography
BV: = Bone Volume
CoCrMo: = Cobalt-chromium-molybdenum
Fe+CoCr: = Female-Cobalt-Chromium
Fe+PE: = Female-Polyethylene
Fe-sham: = Female-Sham
Ma+CoCr: = Male-Cobalt-Chrome
Ma+PE: = Male-Polyethylene
Ma-sham: = Male-Sham
MoM: = Metal-On-Metal
Osteol: = Osteolysis
ROI: = Region of Interest
THA: = Total Hip Arthroplasty
TV: = Tissue Volume
UHMWPE: = Ultra-high Molecular Weight Polyethylene
